# Supplementary material for: Optimized Modeling of Metastatic Triple-Negative Invasive Lobular Breast Carcinoma
Source: Cancers (Basel). 2023 Jun 22;15(13):3299. doi: 10.3390/cancers15133299 (PMC10340059; doi:10.3390/cancers15133299)
Supplement: Supplementary file 1 [file cancers-15-03299-s001.zip › cancers-2416072-supplementary.pdf]

Supplementary Materials

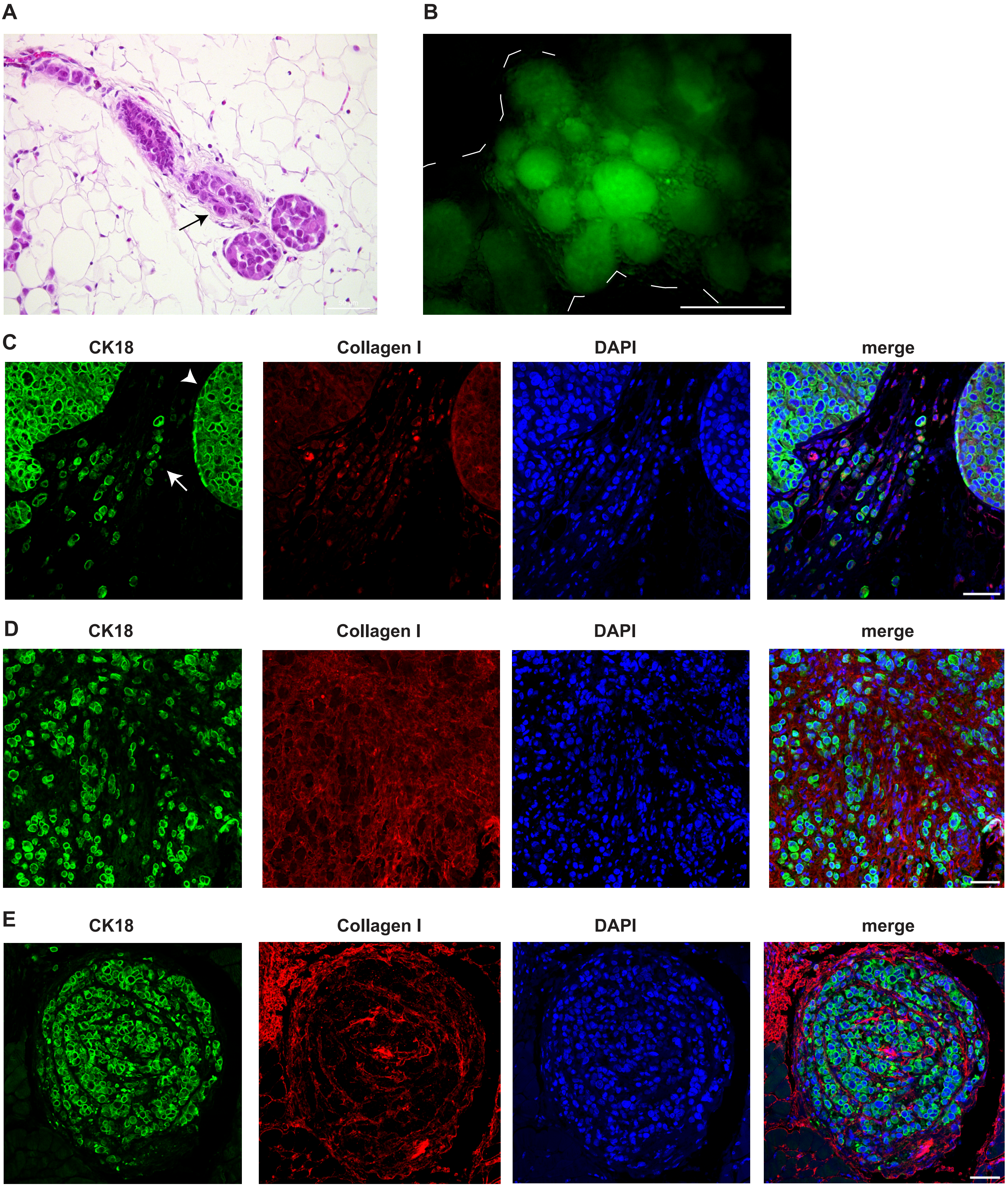

Supplementary Figure S1. Morphological analysis of IPH-926 xenografts.
(A) Representative micrographs of H&E-stained histological section of xenografted mammary gland 1 month after intraductal injection with IPH-926 cells. Scale bar, 50μm.
(B) Fluorescence stereo-micrograph of xenografted mammary gland 4 months after intraductal injection with IPH-926 GFP-luc2 cells. Scale bar 1 mm.
(C) Representative micrographs of triple co-stained histological section (CK8, collagen type I, and DAPI) of xenografted mammary glands 4 months after intraductal injection. White arrowhead points to LCIS, and the arrow points to invasive cells. Scale bar, 50 μm.
(D) Representative micrographs of triple co-stained histological section (CK8, collagen type I, and DAPI) of xenografted mammary glands 4 months after intraductal injection showing extensive invasion. Scale bar, 50 μm.
(E) Representative micrographs of triple co-stained histological section (CK8, collagen type I, and DAPI) of xenografted mammary glands 4 months after intraductal injection showing targetoid spead. Scale bar, 50 μm.
